# Vitamin D Status in Thalassemia Major: an Update

**DOI:** 10.4084/MJHID.2013.057

**Published:** 2013-09-02

**Authors:** Ashraf Soliman, Vincenzo De Sanctis, Mohamed Yassin

**Affiliations:** 1 Departments of Pediatrics, Hamad Medical Center (HMC), Doha (Qatar); 2 Pediatric, Adolescent Outpatient Clinic, Quisisana Hospital, 44121 Ferrara (Italy); 3 Department of Hematology, Alamal Hospital, HMC, Doha, (Qatar)

## Abstract

The survival of patients with thalassemia major has progressively improved with advances in therapy; however, osteoporosis and cardiac dysfunction remain frequent complications. Adequate circulating levels of vitamin D are essential for optimal skeletal health and reducing fracture risk. Vitamin D deficiency and insufficiency is reported to be high in thalassemic patients in many countries despite the presence of good sunshine and routine prescription of 400–1,000 IU vitamin D per day. The risk of vitamin D deficiency in thalassemia and its relation to bone disease; including osteoporosis, rickets, scoliosis, spinal deformities and fractures as well as to cardiac dysfunction is discussed in this mini-review. Monitoring and maintaining normal serum level of 25-OH vitamin D through oral intake of vitamin D and early correction of VDD by oral or parental use of vitamin D may significantly improve bone mineral accretion and ameliorate cardiac function.

## Introduction

Vitamin D (VD) is critical for calcium (Ca) homeostasis and for mineralization of the skeleton, especially during periods of rapid growth, namely infantile and pubertal growth periods. Vitamin D Deficiency (VDD) leads to rickets (a mineralization defect at the epiphyseal growth plates and bone tissue) and osteomalacia (a mineralization defect of bone tissue).^1^

Vitamin D is transported to the liver and hydroxylated to 25-hydroxy vitamin D3(25OH D). Regulated by parathyroid hormone, additional hydroxylation to 1,25-dihydroxyvitamin D3 takes place in the kidney. The major circulating metabolite of vitamin D is serum 25 OHD, which has a half-life of between 10 and 19 days.^2^ It is the best indicator of vitamin D status and reflects levels from dietary intake and synthesis in the skin.^3^ Levels <25 nmol/L (10 ng/ml) are generally considered deficient; levels <80 nmol/L (32 ng/ml) are considered insufficient.^4^ 1,25-Dihydroxyvitamin D3 is the active metabolite of vitamin D that increases intra- and extracellular calcium concentrations by several mechanisms: it absorbs intestinal calcium, diminishes renal calcium excretion and, in conjunction with parathyroid hormone, mobilizes calcium from bone. The initial calcium uptake is the rate-limiting step in intestinal calcium absorption and highly dependent on vitamin D.^5^ 1,25(OH)2D3 involvement in the renal handling of calcium and phosphate continues to be controversial due to the simultaneous effects of 1,25(OH)2D3 on serum PTH and on intestinal calcium and phosphate absorption, which affect the filter load of both ions. 1,25(OH)2D3 enhances renal calcium reabsorption and calbindin expression and accelerates PTH-dependent calcium transport in the distal tubule,^6^ the main determinant of the final excretion of calcium into the urine and the site with the highest Vitamin D receptor (VDR) content. The epithelial calcium channel (ECaC) is an important target in 1,25(OH)2D3-mediated calcium reabsorption. Several putative VDR binding sites have been located in the human promoter of the renal ECaC. Decreases in circulating levels of 1,25(OH)2D3 concentrations resulted in a marked decline in the expression of the channel at the protein and mRNA levels.^7^,^8^

The effect of 1,25(OH)2D3 in improving renal absorption of phosphate in the presence of PTH may not be due to a direct action of the sterol on the kidney

In thalassemia patients bone disease becomes an important cause of morbidity. Problems include osteoporosis, rickets, scoliosis, spinal deformities, nerve compression and fractures.^9^–^11^ Impaired calcium homeostasis is thought to be a consequence of iron overload seen in β-thalassemic transfused patients. Both defective synthesis of 25 OH vitamin D (25OH D) and/or hypoparathyroidism have been described in these patients and negatively affect their bone metabolism.^11^–^15^

Adequate calcium intake and vitamin D administration during skeletal development can increase bone mass in adolescents and decrease bone loss in adult life. In adolescents, there is an inverse relationship between serum 25OH D levels and parathormone (PTH) levels^16^,^17^ and a positive association between serum 25(OH)D levels and bone mineral density (BMD).^16^,^17^

## Epidemiology of VDD in Thalassemia

In North America, Thalassemia Clinical Research Network surveyed 361 patients with thalassemia and reported that 12.8% of the subjects had 25OH D concentrations less than 27 nmol/l and 82% less than 75 nmol/l, regardless of the thalassemia syndrome. Participants with VDD had significantly lower BMD and a positive non-linear relationship was found between 25-OH D and BMD Z-score (adjusted for age) reaching a plateau at 25OH D concentrations >37.5 nmol/l.^18^,^19^

Another report from the USA on 96 patients with thalassemia revealed that 70 (73%) were either deficient (<20 ng/ml, 43%) or insufficient (20–29 ng/ml, 30%). Adolescents had lower 25OH D levels than children and adults.^20^ In Tehran, 37.2% of 220 thalassemic patients had VDD ( < 27 nmol/L).^21^

A report from North India and another from Thailand showed VDD prevalence of VDD 80% and 90% in thalassemic patients, respectively.^22^,^23^

## Vitamin D and Bones

Without vitamin D, only 10–15% of dietary calcium and about 60% of phosphorus is absorbed. The active form, 1,25- (OH)2D3 markedly increases the efficiency of intestinal calcium and phosphorus absorption.^24^–^30^

Serum levels of 25-OHD are directly related to bone mineral density with a maximum density achieved when the 25-OH-D level reached 40 ng/ml or more.^29^ Serum levels below 30 ng/ml are associated with a significant decrease in intestinal calcium absorption. In children, adolescents and adults this is associated with increased PTH and decreased IGF-I.^24^–^34^

PTH enhances the tubular reabsorption of calcium and stimulates the kidneys to produce 1,25-dihydroxyvitamin D.^20^–^25^ It also activates osteoblasts, which stimulate the transformation of preosteoclasts into mature osteoclasts. Osteoclasts dissolve the mineralized collagen matrix in bone, causing osteopenia and osteoporosis and provide enough calcium to prevent hypocalcemia.^35^–^37^

Systemically IGF-I stimulates the production of 1, 25- (OH)2D3 by kidney cells independently of GH.^38^–^41^ IGF-I stimulates bone formation, even in absence of growth hormone (GH), through an intrinsic action on osteoblasts. It supports proliferation, differentiation, and matrix synthesis in cultures of osteoblast-like cells and bone organ cultures and potently stimulates the production of type I collagen (the main structural protein of bone) and increases pro-collagenα1 (I) mRNA expression both in osteoblasts in vitro and in bone in vivo.^42^–^44^

Locally in the growth plate, 1,25-(OH)2D3 potentiates local IGF-I synthesis in chondrocytes and stimulates cell proliferation and differentiation as judged by increased alkaline phosphatase (ALP) activity, collagen X mRNA, and matrix calcification in long-term experiments. 1,25-(OH)2D3 stimulates chondrocytes proliferation and cell differentiation. This proliferative effect is mediated by local IGF-I synthesis.^44^

Consequently, during VDD decreased circulating and locally produced IGF-I in children appears to be a gradual adaptive process to inhibit linear growth (in growth plate) and bone mineral accretion (diaphysis). This process conserves bone minerals and proteins to maintain normal serum Ca concentration and slows down the breakdown of the already formed bones instead of consuming them in forming new bones. In addition, decreased circulating and locally produced IGF-I concentrations can explain in part the defective matrix calcification. The irregular maturation of chondrocytes, and the large irregular hypertrophic zone found in the growth plate of rachitic children can be explained by the prolongation of the cell cycle time, defective maturation and impaired calcification due to the effect of high level of PTH (stimulates proliferation of chondrocytes) in the presence of low IGF-I (delays maturation and calcification of chondrocytes). In addition, low IGF-I may also allow the catabolic effect of PTH necessary for effective release of calcium and explains the osteoporotic appearance of the diaphysis in rachitic children.^24^,^45^

## Pathophysiology of VDD

The development of clinical manifestations of VDD is dependent on many factors including : 1. the severity of the VDD (circulating concentrations of 25-OH-D; 2. the duration of the deficiency; 3. the rate of the child’s growth (which influences Ca demands); 4. the amount of Ca intake and 5. the interaction between bones (osteoblastic and osteoclastic activities) kidneys, gut and important hormones including PTH and IGF-I.^25^–^31^ The latter process entails an important adaptation mechanism that efficiently defend the body against the deleterious effects of hypocalcaemia (PTH increases 1 alpha hydroxylation of vitamin D in the kidney that increases intestinal calcium absorption and increases osteoclastic activity to maintain serum calcium). It is well appreciated that due to this potent adaptation process, the overt cases of rickets and osteomalcia represent only the tip of the iceberg of patients with severe VDD.^8^,^46^

In young children, during the early stages of VDD, decreased intestinal calcium absorption leads to moderate and/or intermittent increase of PTH stimulates the production of 1α(OH)2D3 that increases Ca absorption from the gut, and ensures maintenance of normal serum Ca concentration important for adaptive stage. With longer and/or more severe vitamin D deficiency PTH secretion becomes higher and continuous. This high PTH maintains normal serum Ca concentration on the account of bones (significant osteoclastic activity) and with a significant phosphaturic effect that leads to hypophosphatemia. Secretion of IGF-I decreases further with slowing of bone growth that economize bone use of calcium.^24^–^27^,^46^

Hypophosphatemia further compromises mineralization of the growth plate and bones. This stage is usually associated with significant radiological manifestations. In the late/terminal (dysadapted ) stage, failure of these adaptive mechanisms (low IGF-I, very slow or arrested bone growth and high PTH) and significant exhaustion of bone hydroxyapatite lead to hypocalcemia.^24^,^47^

Adolescents with VDD have better adaptation to VDD compared with young children due to their larger bone mass (Ca and PO4 stores) and relatively slower growth rate (lower requirement for calcium and PO4 per kg ) compared with infants and young children.^46^–^53^

In thalassemic children and adolescents many factors can compromise the adaptation process to VDD including: IGF-I deficiency, hypoparathyroidism due to iron deposition in the parathyroid gland, delayed puberty and hypogonadism, decreased bone mass and decreased synthesis of 25-OH-D,due to hepatic siderosis.^19^,^47^,^54^

Several studies have reported a higher risk of vitamin D deficiency due to genetic and ethno-cultural factors; dark skin or concealing clothing that may lead to limited sun exposure.^19^,^40^–^47^ Decreased outdoor activities in thalassemic patients can also compromise cutaneous synthesis of vitamin D.

A blunting of PTH response to VDD and a combination of hypovitaminosis D and hypoparathyroidism has been described in many thalassemic patients.^48^ Histopathology shows that in suboptimally blood-transfused thalassemics with iron overload.^47^,^55^ Osteopenia is primarily caused by focal osteomalacia as well as decreased bone formation. Densitometric and histomorphometric studies indicated impairment of both trabecular as well as cortical bones in those hemochromatotic patients.^47^

## Clinical Manifestations and Presentation of VDD in Thalassemic versus non-Thalassemic Patients

The clinical spectrum of VDD ranges from subclinical to frank deficiency, with serum 25OHD levels less than 20 ng/dl. This clinical presentation depends on the potent adaptation process that defends the body against hypocalcemia during VDD.^24^,^46^,^53^

In children, the classic clinical manifestations include: delayed linear growth, teething and closure of the fontanel, broad wrist joints, rachitic rosaries, bow legs, Harricon’s sulcus hypotonia and irritability. In severe deficiency hypocalcemic tetany and fractures may occur. The basic skeletal lesion is impaired mineralization of the matrix produced by growth-plate chondrocytes or osteoblasts. Radiological changes include absent or irregular line of ossification at metaphyseal front, excessive osteoid deposition (wide wrist space) with cupping, decalcification of the metaphysis, and the cortex of long bones with subperiosteal erosion of the shafts.^24^–^46^

In adolescents, presentation of severe and/or prolonged VDD markedly differs from young children. They present with vague manifestations including pain in weight bearing joints, back, thighs and/or calves, difficulty in walking and/or climbing stairs and/or running, muscle cramps, facial twitches and carpo-pedal spasms. Adolescents with VDD have higher serum calcium, PO4 and IGF-I concentration and lower PTH and ALP concentrations compared to children with VDD. Radiological changes are less frequent and less significant compared to children with rickets. Radiological manifestations are also less common and may present in the form of pseudo-fractures affecting the femur neck or scapula, generalized or metaphyseal osteoporosis.^46^,^53^

In thalassemic patients symptoms of VDD are commonly confused with the symptoms of anemia and side effects of chelation therapy including: joint and back pain, muscle weakness and osteopenia/osteoporosis. However, improvement of back and joint pains and increased tolerability for walking and exercise has been reported in thalassemic adolescents after treatment with vitamin D.^47^,^53^ Even in adequately treated thalassemic patients, some radiological abnormalities of the long bones and vertebrae appear similar to those described in rickets, especially after long-term chelation therapy.^57^

## Vitamin D Deficiency and Heart in Thalassemia Major

Low vitamin D is linked to decreased cardiac function, muscle weakness, glucose insensitivity and refractory congestive heart failure. An ejection fraction less than 56% is considered abnormal and indicates poor pump function.^56^,^57^

In one study in 24 thalassemic patients, there was a proportional association between low vitamin D, high cardiac iron and increased ventricular dysfunction.^57^ However, causation has not been proven. Vitamin D screening and replacement are probably indicated regardless of the heart findings.

In addition, low vitamin D produces secondary hyperparathyroidism, which exacerbates heart failure of any etiology. Both parathyroid hormone and vitamin D1-25OH appear to stimulate transmembrane calcium movement via L-type voltage-dependent calcium channels (LVDCC).^56^ In addition, elevated serum parathormone levels are associated with myocardial iron overload in patients with beta-thalassaemia major.^58^

The association of vitamin D deficiency with left ventricular dysfunction is not surprising. Skeletal muscle weakness and chronic heart failure exacerbation have been well described with vitamin D deficiency.^59^,^60^

Murine data indicate that LVDCC are important in transporting non-transferrin bound iron (NTBI) into the myocardium.^62^ Thus LVDCC modulation represents the logical link between vitamin D deficiency, cardiac iron, and cardiac function. Because of this, thalassemia patients especially those with ventricular dysfunction should have their vitamin D levels assessed, and replacement should be started if these levels are low.^56^,^57^,^61^

## Treatment

Identifying the optimal strategy for replacing vitamin D in patients with thalassemia is critical because adequate circulating levels of vitamin D are essential for optimal skeletal health and reducing fracture risk.^20^

There is mounting evidence that, in the absence of adequate exposure to sunlight, 1000 IU dietary or supplemental vitamin D2 per day is required in adults to prevent vitamin D deficiency. To maintain a healthy blood level of 25-OH-D, most healthy patients require at least 1000 IU of vitamin D2 each day if they do not get exposure to the sun and there is evidence that doses up to 2000 IUper day can be considered safely.^62^–^65^

Recommended repletion therapy consists of 50 000 IU of vitamin D2 weekly for 8 weeks or 2000 IU of vitamin D3 daily for 8 weeks.^65^ Although this 8 week- course of cholecalciferol (total of 400,000 IU) supplementation can normalize mean serum 25-OH-D and PTH levels in patients with chronic hypovitaminosis D, however, such supplementation could not maintain their 25-OHD levels in the sufficient range for 1 year.^66^

Recent data demonstrated that the problem of inadequate vitamin D status in thalassemia has persisted despite routine daily supplementation of 400–1,000 IU vitamin D. The intermittent high-dose supplementation with oral 50,000 IU vitamin D2 every 3 weeks proved to be an effective and safe strategy for increasing 25-OH D levels. Administration of each dose of 50,000 IU vitamin D2 increased serum 25-OH vitamin D by 1.4 ± 2.0 ng/ml. Regardless of the baseline vitamin D level or the duration of the supplement regimen, no 25-OH D level >80 ng/ml was observed over the course of the observation period. The rate of decline was on an average 1.5 ng/ml per month. Hence, patients who had inadequate vitamin D status on screening were likely to require ongoing high-dose supplementation.^20^

Another group has studied the efficacy of high-dose vitamin D3 (10,000 IU/kg) in thalassemia given as a single intramuscular injection. Although a majority of the patients showed an improvement in 25-OH D level to >20 ng/ml, the effect did not persist at 6 months.^47^ Therefore it is recommended to give another IM mega-dose of vitamin D or give oral vitamin D maintenance to these patients after 3 months of the first injection in order to keep their 25 OH D level in the acceptable range.

## Recommendations of Vitamin D Intake in Thalassemic Patients

Patients with thalassemia should have adequate vitamin D status to assure healthy bone accretion. The authors recommend the following:

Vitamin D status shall be assessed annually in all children and adults with thalassemia.^20^ Those with serum 25 OH vitamin D below 20 ng/ml (50 nmol/L) should be treated with vitamin D. Repletion therapy consists of 50 000 IU of vitamin D2 weekly for 8 weeks or 2000 IU of vitamin D3 daily for 8 weeks. For maintenance we recommend daily vitamin D oral intake of 800–1000 units or 50. 000 IU per month or a mega dose of vitamin D (10.000 U/kg, maximum 600,000 IU) every 6 months (either orally or intramuscularly) especially those who do not receive adequate sun exposure. (Table 1) We also recommend that 25 OH D levels should be monitored every six months in patients on high-dose supplementation to ensure adequacy of therapy and to monitor toxicity.

## Vitamin D Supplementation on BMD

To date, three studies are available in literature on the favourable effects of vitamin D on bone density. In a study done at Cukurova region, in thalassemia patients having normal levels of 1,25(OH)2D3, the effects of zoledronate (once in every 6 months, 4 mg i.v.) and calcitriol (0.25 mcg/day) treatments were compared. In zoledronate arm, lumbar BMD; and in calcitriol arm femur neck BMD improved significantly. The difference for BMD and T scores in lumbar and femoral neck was not significant between groups.^67^

Leung et al. followed 39 thalassaemia major patients over a 3 years period. BMD values significantly improved only in those treated with monthly pamidronate in addition to standard treatments with calcium and vitamin D.^68^ In another study performed by Patiroglu et al.^69^ the thalassaemic treated group received 15 mg pamidronate infusion every 3 months for one year. The next year all patients received only calcium and vitamin D supplements. After two years, femoral neck and lumbar spine BMD significantly increased compared to baseline.

## Conclusions

In conclusion, high prevalence of vitamin D deficiency occurs in thalassemic children and adolescents that may largely contribute to their bone diseases. Monitoring normal serum level of 25-OH D through oral intake of vitamin D and early correction of VDD by oral or parental use of vitamin D may significantly improve their bone mineral accretion and prevent bone disease.

## Figures and Tables

**Table 1:** Recommendation for vitamin D assessment and therapy in patients with thalassemia.

Assessment of vitamin D and therapy	Frequency/dose
**Serum 25OH vit D 3 status shall be measured in all children and adults with thalassemia.**	Annually or Biannually in patient on mega dose therapy
**For thalassemic patients with 25OH D3 level < 20 ng/ml should be repleted as following:**	50 000 IU of vitamin D2 orally weekly for 8 weeks or 2000 IU of vitamin D3 orally daily for 8 weeks or a mega dose of 10.000 IU/kg (max 600.000 IU) orally or IM once.
**For thalassemic patients with 25OHD3 level > 20 ng/ml maintenance therapy can be given especially in places with poor sun exposure as following:**	800–1000 IU of vitamin D2 orally daily or 50. 000 IU of vitamin D2 orally per month or a mega dose of vitamin D (10.000 IU/kg, maximum 600,000 IU) orally or IM every 6 months.
